# Perceptions of Sports Betting and Promotions in the U.S.: Evidence from a Mixed-Methods Sentiment Analysis of YouTube Comments

**DOI:** 10.1007/s10899-025-10467-y

**Published:** 2026-01-19

**Authors:** Kwangmi Kim, Kyungeun Park, Kyongil Yoon, Yanggon Kim

**Affiliations:** 1 Department of Mass Communication, Towson University, Towson, MD 21252 USA; 2 Department of Computer Studies, Notre Dame of Maryland University, Baltimore, MD USA; 3 Department of Computer & Information Sciences, Towson University, Towson, MD USA

**Keywords:** Social media and gambling, Sports betting, Advertising and promotion, Public perceptions, Sentiment analysis, YouTube comments

## Abstract

This study examines public attitudes toward sports betting and promotions in the United States by analyzing YouTube comments. Using keyword searches, 210 videos were identified, yielding 2,369 comments for analysis. A hybrid approach combining automated BERT sentiment analysis with human-coded content analysis was employed to determine overall sentiment and to identify key themes, endorsers, and sportsbook brands. Results revealed a predominance of negative and neutral sentiments, with positive sentiments least frequent. BERT detected more negative sentiments than the human-coded analysis, which indicated a more balanced distribution. Content analysis showed that positive responses were often directed at entertainment features such as humor, music, and celebrities, whereas negative responses focused on celebrity endorsements, excessive advertising exposure, and frustrations with sportsbook brands’ customer services. Prominent celebrities, including Charles Barkley and Paige Spiranac, received relatively favorable comments, while Wayne Gretzky and Jamie Foxx were criticized. Larger sportsbook brands such as BetMGM, DraftKings, and FanDuel were mentioned most often but attracted more negative feedback compared with smaller brands. These findings underscore the need for advertisers to balance entertainment with responsibility, for sports betting operators to improve transparency and user experience, and for regulators to establish clearer guidelines on advertising practices. Future research should consider longitudinal approaches and extend analysis to additional social media platforms to capture evolving public attitudes toward sports betting.

Sports betting had been banned in the U.S. since October 1992 by the Professional and Amateur Sports Protection Act (PASPA). However, the U.S. Supreme Court overturned this Act in 2018 and gave each state the right to legalize sports betting. As of July 2025, sports betting is legal in 39 states and Washington D.C. with mobile sports betting allowed in 31 states (Yakowicz, 2025). Due to the relatively young history of legalized sports betting in the U.S., academic research on sports betting is still in its early stages. Given the current scarcity of research in the U.S., it is important to gauge the current perception of sports betting and promotions in the U.S. The main objective of this study is to identify and analyze overall sentiments reflected on social media and gauge public opinion on sports betting, focusing on the roles of endorsers and sportsbook brands. A hybrid method by integrating automated computer analysis with human-coded analysis is used for the analysis.

## Background

### The Growth of Sports Betting in the U.S. And Advertising Spending

There are no regulations on sports betting promotions in the U.S. at the federal level. Each state has its own regulation on sports betting, but few regulations on promotions. Most states require sportsbooks ads to display a legal gambling age and a “warning label” against addiction with a gambling hotline phone number (J. Hernandez, 2022). According to the American Gaming Association (2024), sportsbook operators, excluding fantasy sports, spent about $1.2 billion in 2023 and $1.4 billion in 2022 on advertising. TV was the most popular medium (taking about 80% of media voice), followed by digital and radio. FanDuel, DraftKings, Wynn, MGM, and Caesars spent $25.6 million on streaming, display, and social advertising in January 2022 (Sramek, 2024). These five brands control at least 82% of the market in each state. The AGA reported that Americans wagered an estimated $150 billion in 2024, a $30 billion increase over 2023 with sports betting operators generating more than $14 billion from these wagers, up 27% from 2023 (Yakowicz, 2025). Sports betting in the US is expected to grow, with Goldman Sachs Research predicting it could bring in around $45 billion annually once the market matures (Barwick, 2024).

According to the Pew Research Center, about half of U.S. adults (56%) were aware of the legalization of sports betting and 19% of U.S. adults had bet money on sports over a year through various forms, either in person at gambling venues, or online with betting apps in 2022 (Gramlich, 2022). Most Americans had no strong positive or negative opinions (57%) but one-third of U.S. adults (34%) recognized the harmful aspects of sports betting, while few saw it as beneficial for society (8%) or for sports (16%). The Pew survey also indicated that there were differences in sports betting by gender and race: More men than women (24% vs. 15%) and more Black (27%) and Hispanic adults (24%) than White (18%) and Asian Americans (10%) participated in sports betting, while there were no significant differences by education, income, or political party affiliations (Gramlich, 2022).

### The Literature on Sports Betting Promotions: Topics

The literature on sports betting, primarily from the United Kingdom and Australia, covers various topics such as the effects of sports betting advertising on betting behaviors and gambling (Hing et al., 2024; Vieira et al., 2023), regulatory issues (Holden, 2019; Petrotta, 2023), and psychosocial or cultural factors associated with sports betting (Li et al., 2020; Lin & Lu, 2015). Other areas of focus include content analysis of sports betting advertising and promotions (Killick & Griffiths, 2023), the perception and attitudes towards sports betting (Seal et al., 2022; Youn et al., 2000), and the impacts of sports betting advertising on these perception and attitudes (Deans et al., 2017; Lamont et al., 2016; Lopez-Gonzalez et al., 2020).

Research on the impact of sports betting advertising shows that promotional inducements or incentives, such as cashback offers and bonus bets, have prompted more people to engage in sports betting and lowered their perceptions of the associated risks (Deans et al., 2017; Killick & Griffiths, 2022; Lopez-Gonzalez et al., 2020). Also, using celebrities and humorous approaches has contributed to the normalization of sports betting by presenting gambling as harmless, fun, and successful (Lamont et al., 2016).

Sports betting advertising does not affect everyone in the same way. Various demographic and behavioral factors play a role in shaping perception and attitudes. Men, particularly young men, sports bettors, and minority groups tend to view sports betting advertising more positively than women, non-bettors, and white people. Particularly, the constant and over-exposure to sports betting advertising have led to more frequent discussions about sports betting among young men, making them perceive sports betting as part of sports (Deans et al., 2017; Pitt et al., 2016; Thomas et al., 2012).

## Research Questions

This study does not aim to establish causal relationships between sports betting advertising and attitudes toward sports betting. Instead, it seeks to understand public perceptions of sports betting and advertising, and to identify key elements that attract audience attention by analyzing user comments on sports betting commercials. Drawing on prior research on sports betting promotions, celebrity endorsements, and consumer sentiment, the following research questions were developed:

1. What is the overall sentiment towards sport betting and advertising in the U.S.?
2. How do people perceive celebrities’ roles in sports betting promotions?
3. Which celebrities received the most attention from the audience?
4. What are people’s responses to sportsbook brands?

## Methods

YouTube has been recognized as a platform that serves as a vibrant forum for public engagement and discussion (Fares et al., 2024; Snelson, 2011; Teng et al., 2020). By reading comments, posting replies, or sharing their opinions and information on a topic, YouTube users actively engage with their topic of interest. To gauge the attitudes and perceptions of sports betting and promotions in the U.S., this study analyzed YouTube comments on these commercials by using automated analysis along with human-coded content analysis. This hybrid approach allowed us to gain a more in-depth understanding and is relevant as self-reported research methods like surveys often raise questions about insufficient and inaccurate responses to sensitive topics (Killick & Griffiths, 2023; Yu et al., 2020).

To collect user comments, a video search was conducted in July 2024 to identify sports betting commercials on YouTube. Using YouTube’s keyword search function, we employed the terms “sports betting commercials,” “DraftKings,” “FanDuel,” “Fanatics/PointsBet,” “BetMGM,” “Caesars,” and “Bet365.” These brand names were selected because multiple industry sources consistently identified them among the top ten sportsbook operators in the United States in 2024 (Duchesne, 2024; Kritzer, 2024; Sciangula, 2024). Including specific operator names in the search yielded a greater number of relevant advertising videos than using general keywords such as “sports betting commercials.*”* Although the search was based on a predefined list of major sportsbook brands, user comments occasionally mentioned other operators that were not part of the initial search keywords, as some viewers referenced or compared their own experiences with different brands.

A video was considered a sports betting commercial if it was a professionally produced advertisement promoting a licensed sportsbook operator or its products/services. Videos that were news clips, commentary or reaction videos, user-generated content, “shorts,” or unrelated materials were excluded. This process resulted in 210 unique videos, each of which was included regardless of its popularity or posting date. User comments from these 210 videos were then scraped using YouTube’s API in Python, yielding a total of 2,398 comments. After removing duplicates and non-English entries, 2,369 comments were retained for analysis.

First, all comments were categorized according to their relevance to the topic of commercials. They were grouped into three categories: commercial-related comments, betting-related comments, and non-relevant (“Other”) comments. A comment was coded as commercial-related when it directly referred to the content, style, tone, or message of a commercial—for example, discussing the ad’s creativity, featured athletes or spokespersons, or brand appeal. Comments that focused on sports betting activities, such as odds, wagers, or betting outcomes, were coded as betting-related. Comments that did not fit either of these two areas were classified as Other/Not relevant. These were typically one-word responses, symbols, emoticons, or replies lacking sufficient context for meaningful interpretation. Therefore, they were excluded from further analysis. All categories were defined as mutually exclusive, with each comment assigned to a single category based on its dominant topic of discussion. This classification framework was adapted from previous research that examined comment relevance in online discussions (Madden et al., 2013) and was operationalized here to fit the specific context of commercial- and betting-related discourse.

Then, sentiment analysis was conducted for commercial-related comments as well as for betting-related comments, respectively. The sentiments of the comments were found in impressions, opinions, or expressions of personal feelings (Deori et al., 2023; Madden et al., 2013) with three categories: Positive, negative, and neutral. Typically, words like “like,” “love,” “awesome,” “great,” “funny,” or “exciting,” are associated with positive sentiments while words like “hate,” “disgusting,” “pathetic,” or “terrible” are associated with negative sentiments. Sentiment analysis was conducted by two methods: Bidirectional Encoder Representations from Transformers (BERT) analysis as well as traditional human-coded content analysis. Using both approaches allowed us to evaluate the reliability of automated machine learning–based sentiment detection in comparison with human judgment. It also helped us determine whether BERT accurately captured audience sentiment in this context.

The BERT analysis is a deep learning model developed by Google in 2018 for natural language processing tasks and is considered strong as it processes the comments bi-directionally, unlike traditional static word-based models (Devlin et al., 2019)). Three pre-trained transformer models (FinBERT, TweetEval, and DistilBERT) were formed to make BERT-Ensemble model for this analysis. FinBERT is specialized on processing financial texts, making it particularly effective in analyzing sentiment in stock market reports or financial news. TweetEval is optimized for social media sentiment analysis, specifically designed to handle short, informal, and contextually nuanced texts such as tweets and user-generated comments. DistilBERT, a compressed and computationally efficient variant of BERT, serves as a general-purpose sentiment classification model.

Traditional human-coded content analysis was also conducted to gain in-depth understanding and to verify automated analysis’ findings. Three researchers were involved in coding as two researchers worked as a team on a rotating basis (i.e., coders A and B, coders B and C, and coders C and A) to code 100 sample comments and calculate inter-coder reliability. Cohen’s Kappa values for relevance were 0.878, 0.913, and 0.862, while values for sentiments were 0.855, 0.838, and 0.889, indicating high coding reliability (McHugh, 2012).

## Findings

As described in the method section, it was first necessary to determine how many comments were related to the study topics. Approximately 67% of the total comments (1,577) were relevant, addressing either commercials (1,441) or betting experiences (136), while 33% (792) were not related to either category. Commercial-related comments significantly outnumbered betting-related comments. Comments categorized as “Other/Not relevant” were typically one-word responses, symbols, emoticons, or replies lacking context. Because these could not be meaningfully assessed, they were excluded from further analysis.

### Sentiment Analysis

To address the first research question, sentiment analysis was conducted using two complementary approaches applied to the entire dataset. According to the BERT analysis, among the 1,577 relevant comments, negative sentiments (*n* = 632) outnumbered neutral (512) and positive (433). By contrast, the human-coded content analysis indicated that neutral comments (*n* = 584) slightly outnumbered negative (549) and positive (444). Human-coded results suggested smaller margins between neutral and negative sentiments compared with BERT. Both approaches, however, found that positive sentiments were the least frequent. Positive sentiments were most often directed toward features of the commercials, such as music and favorite celebrities, whereas negative sentiments were primarily associated with celebrity endorsements, excessive advertising exposure, and users’ relationships with sportsbook brands, often in relation to concerns about gambling addiction.

We also examined whether sentiment patterns differed between commercial-related and betting-related comments. Among commercial-related comments (*n* = 1,441), BERT detected a greater proportion of negative sentiments compared with the human-coded analysis, making negative sentiment the most prevalent category, followed by neutral and positive sentiments. In contrast, the human-coded analysis found neutral sentiment to be the most prevalent, followed by negative and positive sentiments. This pattern was consistent with the overall dataset. For betting-related comments (*n* = 136), both BERT and human-coded analyses identified negative sentiment as predominant, followed by neutral and positive sentiments, with positive comments being the least frequent (see Table 1).

**Table 1:** Number of comments by sentiment (Human-coded analysis and BERT analysis)

Category	Positive	Negative	Neutral	Total	Positive	Negative	Neutral
All relevant comments	444	549	584	1,577	433	632	512
Commercial-related comments	422	489	530	1,441	414	556	471
Betting-related comments	22	60	54	136	19	76	41

Note. Human-coded and BERT analyses represent the number of comments classified as positive, negative, or neutral within each category. Totals reflect all relevant comments (N = 1,577)

A chi-square test of independence indicated that the sentiment distributions differed significantly between the two methods, χ² (2, *N* = 3,154) = 10.70, *p* <.01. This finding suggests that although both methods revealed similar overall patterns, BERT tended to classify comments as negative more frequently and as neutral less frequently than human coders. The dual analytic approach offers both practical and methodological value: while BERT enables quick sentiment detection, human coding captures subtle cues that the automated model may miss.

### Celebrities: Roles and the Perceptions

Among the 1,441 comments related to commercials, the most prominent topic concerned celebrities (695 comments), followed by humor and entertainment (303), frustration with commercials (165), and concerns about gambling and gambling problems (51). The prominence of celebrity-related discussion led us to focus on how commenters perceived celebrities and their roles in sports betting promotions.

The analysis of celebrity-related comments (695 comments) focused on their popularity and attention-getting potential. A total of 84 individuals were mentioned in the comments, although some references to celebrities did not specify a name (332 comments). All endorsers featured in sports betting advertisements were professional athletes, comedians, or actors. Of the 84 individuals, 18 were mentioned more than five times. Table 2 presents the ten most frequently discussed celebrities (or ‘brand ambassadors’) along with the perceptions they received.

**Table 2:** Top 10 celebrities in sports betting promotions and their perceptions

Celebrity	Endorsing Brand	Mentions (*n*)	Positive	Negative	Neutral	Mean Sentiment Score (0–2)
Charles Barkley	FanDuel	33	8	2	23	1.18
Wayne Gretzky	BetMGM	33	8	20	5	0.64
Jamie Foxx	BetMGM	26	2	18	6	0.38
Kevin Hart	DraftKings	20	8	9	3	0.95
Paige Spiranac	PointsBet	19	8	1	10	1.37
Shaquille O’Neal	DraftKings and PointsBet	18	5	10	3	0.72
Kevin Garnett	BetMGM	10	3	0	7	1.30
John Paul Tremblay (for Julian)	PointsBet	8	2	0	6	1.25
John Goodman	FanDuel	7	1	1	5	1.00
Tom Brady	BetMGM	7	1	3	3	0.71
Unspecified celebrities	—	332	99	107	126	0.98
Multiple celebrities	—	28	3	4	21	0.96

Note. Mean sentiment score was calculated by coding each comment as positive (2), neutral (1), or negative (0); higher values indicate more positive perceptions

Charles Barkley (for FanDuel) and Wayne Gretzky (for BetMGM) received the highest number of mentions, followed by Jamie Foxx (for BetMGM), Kevin Hart (for DraftKings), Paige Spiranac (for PointsBet) and Shaquille O’Neal (for DraftKings and PointsBet). Of these, Charles Barkley, Paige Spiranac, and Kevin Garnett were evaluated most favorably, receiving a greater proportion of neutral or positive comments. Many users commented on Barkley’s professional record, his “humble” acknowledgment of never winning a championship, and his humorous roles in commercials. Some commenters noted that, “This is hilarious! I laugh every time I see it. Chuck is one of my favorites,” and “Lol!!! Leave it to Chuck Barkley to make fun of his not winning a title lol!!!”

In contrast, Wayne Gretzky and Jamie Foxx received the greatest proportion of negative comments, with Foxx emerging as the least favorable endorser. Comments about actor Jamie Foxx, for instance, were sharply critical, with one user writing, “Foxx is just cashing a paycheck and he clearly doesn’t care about betting,” while another mocked his BetMGM advertisement by writing, “Step one pretend Jamie Foxx isn’t annoying. Step two try to forget how much of a douchebag Jamie Foxx is. Step three prepare to make a Jamie Foxx voodoo doll. YouTube users criticized him as “greedy,” “shameful,” and “sick” for promoting gambling for BetMGM. Similarly, hockey legend Wayne Gretzky’s participation in betting promotions was described as “disappointing” and noted as “a bad look for someone who used to stand for integrity in sports. Why would the Great One even consider doing a betting app commercial of all things?” As shown in Table 2, several high profile figures including Wayne Gretzky, Jamie Foxx, Shaquille O’Neal, and Tom Brady, received more negative than positive comments. Commenters who expressed negative sentiment perceived that celebrities were involved in sports betting promotions mainly for their financial gains. Table 3 presents illustrative comments for seven well-known endorsers across each sentiment category. The comments on Tom Brady, “no respect for Tom. He doesn’t have enough money?” or “Disgusting that he has to lower himself for this” reflect typical sentiments among commenters. These reactions suggest that perceptions of celebrity endorsements depended less on the product itself than on how authentic and appropriate the endorser seemed for a gambling promotion.

**Table 3:** Examples of comments on selected celebrity endorsers by sentiment category

Celebrity Endorser	Positive Comment Example(s)	Neutral Comment Example(s)	Negative Comment Example(s)
Charles Barkley	This is hilarious! I laugh every time I see it. Chuck is one of my favorites.	Who should Charles Barkley bet on during the 2023 NBA playoffs? Deepfake young Charles is crazy.	Deepfake young charles is crazy.
Wayne Gretzky	Every Gretzky BetMGM commercial has the potential for greatness… and this one totally delivered!	Wayne Gretzky, the Great One, the best hockey player ever and one of the best athletes of all time.	Been a Gretzky fan since his WHA days… and frankly this is a bit disappointing; why would The Great One even consider doing a betting app commercial?
Jamie Foxx	He seems like a good guy. Good to see Jamie back in action.	I am concerned about Jamie Foxx. No one has seen him in months… I truly hope he is ok.	Step one pretend Jamie Foxx isn’t annoying. Step two try to forget how much of a douchebag Jamie Foxx is. Step three prepare to make a Jamie Foxx voodoo doll.
Paige Spiranac	Paige Spiranac is so hot! You know what? Paige IS freaking gorgeous. Paige is such a smokeshow!	That’s Paige Spiranac. Is her name Paige? What’s the last name?	
Shaquille O’Neal	You are a very smart man, Shaq—great stuff. You’re a top bloke; needs to be more of you. And well done for helping out these fellas. They are going to go a long way in life; they deserve it.	32 being Shaq’s playing number for the Magic and he said “flatten ya.”	I thought Shaq was cool. He should be ashamed of himself. Shaq is worth half a billion dollars; why is he promoting gambling companies which help ruin so many people’s lives? How greedy is he? Shaq has lost all respect for doing this ad.
Tom Brady	Tom Brady is the GOAT.	Brady has indeed won enough	No respect for Tom. He doesn’t have enough money?

Note. Examples reflect user-generated YouTube comments about celebrities featured in sportsbook commercials. Comments are quoted verbatim and may contain spelling or grammatical errors. Sentiment balance varies by individual; some endorsers did not have identifiable comments in all sentiment categories like Paige Spiranac

When multiple endorsers were featured, the Manning family stood out. All four members (Archie, Peyton, Eli, and Cooper Manning) appeared in Caesars sportsbook promotions, which attracted predominantly negative and critical feedback. One commenter put a long comment:

Do you feel good about promoting gambling when the Manning family never does it themselves? Do the Mannings teach their kids to gamble their money? Or do they simply not understand the concept of not having money to throw away gambling because they have always been wealthy… How about the jester black guy performing for the Mannings, does he think gambling will help the black community? Pathetic what society accepts; preying on what little people have, knowing they will gamble it all for the 0.0001% chance at a percentage of what these celebrities make.

Overall, the endorsers mentioned in the comments were predominantly male, with only one female celebrity represented among the ten most frequently discussed: former professional golfer Paige Spiranac. Unlike many of her male counterparts, Spiranac received more favorable than unfavorable evaluations (8 positive, 1 negative, and 10 neutral comments). However, her appearances in PointsBet sportsbook promotions drew attention primarily for her physical attractiveness, as reflected in comments such as “Paige Spiranac is so hot,” or “Paige is such a smokeshow!” These comments suggest that while Spiranac generated higher levels of positive engagement, the nature of that attention was often gendered.

The prominence of male figures and the gendered framing of responses to Spiranac reflect broader patterns in sports media and advertising, where women are often valued for appearance rather than athletic or professional credibility (Granleese & Shen, 2009; Pitt et al., 2024; Vai et al., 2025). Similarly, research on gender bias in language and vision datasets shows how representational inequities persist across domains: “a man with a tennis racket is a tennis player. A woman with a tennis racket is just a woman with a tennis racket” (Harrison et al., 2023, p. 5). These parallels mirror the patterns observed in user comments about Spiranac and other female endorsers. Taken together, the findings imply a continuing imbalance in gender representation in sports betting advertising. This imbalance highlights the need for future research to examine how gender and celebrity image interact in shaping public perceptions of sports betting promotions and to consider the implications of representational inequality in gambling-related marketing (Han & Viswanathan, 2025).

### Sportsbook Brands

Sportsbook brands also emerged as an important topic within the YouTube comments, reflecting viewers’ attention to specific companies and their reputations. Out of a total of 1,577 comments, 63 (35 from commercial-related comments and 27 from betting-related comments) specifically mentioned sportsbook brands. BetMGM (23 comments) was the most frequently mentioned brand, followed by DraftKings (14 comments) and FanDuel (14 comments). Four other brands (e.g., Bet365, Barstool, PointsBet, and Caesars) were mentioned less frequently but tended to receive more positive evaluations than the top three brands (see Table 4). It should be noted, however, that the prominence of these brands in the comments partly reflects the inclusion criteria for video selection, as several search keywords contained their names.

**Table 4:** Number of mentions and sentiment of sportsbook brands

Sportsbook Brand	Total Mentions (*n*)	Positive	Neutral	Negative	Mean Sentiment Score (0–2)
BetMGM	23	5	5	13	0.65
DraftKings	14	5	3	6	0.93
FanDuel	14	5	4	5	1.00
Bet365	4	1	2	1	1.00
Barstool	2	2	0	0	2.00
PointsBet	3	2	0	1	1.33
Caesars	3	1	2	0	1.33

Note. Mean sentiment score was calculated by coding each comment as positive (2), neutral (1), or negative (0); higher values indicate more positive evaluations

Another notable aspect is that these three brands (BetMGM, DraftKings, and FanDuel) are not only the most frequently mentioned in the comments but also the top three advertisers among U.S. sportsbook operators. In 2023, FanDuel reportedly spent approximately $157 million on television advertising, DraftKings $123 million, and BetMGM $45 million (Bruell, 2024). Their strong presence in the dataset thus likely reflects both their substantial advertising investment and the inclusion criteria used for video selection rather than purely organic viewer discussion.

Commenters often compared sports betting operators in terms of reliability, promotional offers, and customer service quality, indicating that brand perception extended beyond advertising appeal to broader assessments of trust and user satisfaction. Negative remarks were often directed at the leading brands, particularly regarding customer service and misleading promotions. One viewer complained, “DraftKings keeps changing the bonus rules—it’s impossible to trust them,” while another wrote, “FanDuel takes forever to process withdrawals; they make it easy to deposit but hard to get your money back.” Comments about BetMGM similarly described “too many glitches and not enough support when things go wrong.”

By contrast, positive sentiments appeared more often for smaller or less dominant brands. Users praised their efficiency and responsiveness: “Caesars has been great—fast payouts and easy to reach support,” one comment read, while another said, “PointsBet actually honors their promos, unlike the others.” Table 5 provides illustrative examples of user comments on sportsbook brands and betting apps. Collectively, these comments suggest that trust, transparency, and responsiveness were central to users’ evaluations of sportsbook brands, shaping sentiment more strongly than advertising exposure itself. In particular, users’ perceptions seemed to be guided by their direct experiences with customer service and payout reliability rather than by the frequency or visibility of brand promotions.

**Table 5:** Examples of representative comments by sportsbook brand

Brand	Positive Comment Example(s)	Neutral Comment Example(s)	Negative Comment Example(s)
BetMGM	OMG. Don’t overthink it. Connect the dots. Sportsbooks are for watching sports and placing your bet(s). This sportsbook is MGM; hence BetMGM. It’s not complicated.	Y’all should credit me 100k to my bet mgm account i’ll promo my plays on Instagram I lost all of my parlays last year	Betmgm. Rich mofo’s just wanting to take your money. Fuck off. I’m done with betmgm. Who the heck give out 1 dollar free play after spending a thousand dollars? I hope y’all go out of business. Y’all of the devil. BET MGM IS A SCAM. ༢༥༴ ༭༧༭ ༡༰༰༩ S༭༡༫༩༮༧ ༭༥༩༮༳༡༮༥༳༯༭༯༮༥༰༬༥༡༳༥༨༥༬༰༭༥
DraftKings	Draft king sports book. Good lines, many line options, fair book payout ratios. Easy deposit and withdrawal.	Join DraftKings and make a lineup today. Who’s gonna play him? An employee from DraftKings.	DraftKings stole my money and locked my account after 3 years of winning! I’d never use DraftKings. Only the Barstool sportsbook.
FanDuel	Make the Cornhole Prodigy a FanDuel regular. Have him winning impossible parlays and dancing to the bank. This stranger, a “Cornhole Prodigy,” is the greatest humorous advertisement since Dos Equis. I absolutely howl at this guy.	I’ll deposit one more time to try out the new FanDuel.	Kid me too I hate football people who use FanDuel do not have real lives and waste their lives away. Depressing and a bit blasphemous. Will make a point to avoid FanDuel.
Barstool	I’d never use DraftKings. Only the Barstool sportsbook. No soul. Use Barstool Sportsbook, more entertaining.		
PointsBet	Wish Points Bet would work on the West Coast—the voters here are lame as fuck.		Points Bet sucks. It’s a robbery.
Caesars	Caesars has been great—fast payouts and easy to reach support.	Caesars Sportsbook and Casino NEWEST TV commercial with Cooper Manning, B.J. Smoove, Vince Vaughn.	Yeah it’s the worst commercial of all time because why would Caesar look like him? How dare they! How you turn Julius Caesar into a black man. This makes no sense.
Bet365	I love Bet365.	Bet365 isn’t in Kenya. I think, instead of curling, the sporting event Bet365 is making popular ought to be women’s professional hockey. LOL.	Message to Bet365! BRING BACK RAY WINSTONE!!!
Other/General Comments			I spent 900 dollars on their app; you know what my free play was? It was 1 dollar. They look at us like scum. The whole thing is rigged. Nobody will ever hit the jackpot. The house will always win.

Note. Examples reflect user-generated YouTube comments about sportsbook brands or betting apps. Comments are quoted verbatim and may contain spelling or grammatical errors. Sentiment balance varies by brand; some brands did not have identifiable neutral comments (e.g., Barstool and PointsBet)

A small subset of user comments also expressed personal distress or frustration associated with betting outcomes, including financial loss, compulsive use, or feelings of regret. While these comments were not widespread, their presence suggests that gambling-related harms are part of public discussions surrounding sports betting promotions. Such expressions often carried a tone of self-awareness or criticism, echoing broader concerns about the normalization of gambling behavior in sports media.

## Discussion and Conclusion

This study aimed to examine public perceptions of sports betting in the U.S. by analyzing YouTube comments. Using a hybrid approach that combined automated BERT sentiment analysis with human-coded content analysis, the study provided an in-depth understanding of sentiments and themes reflected in online discussions of sports betting and promotions. By using these combined approaches, this study also offers methodological insights regarding sentiment analysis. Previous work on sentiment analysis was done by using automated programs without clearly verifying their accuracies (Birjali et al., 2021; Singh et al., 2020). This study revealed that while automated models such as BERT enable efficient large-scale sentiment classification, their results were different from human-coded findings. These differences suggest that BERT alone may not yet capture the nuanced emotional tone of user-generated comments on gambling-related content. Therefore, combining automated and human-coded approaches provided a useful validity check, illustrating both the potential of machine learning tools and the continued value of human interpretation in context-sensitive analyses like sports betting.

Overall, the sentiment toward sports betting promotions was negative and neutral sentiments, outnumbering positive ones. While entertainment elements such as music, humor, and celebrity appearances elicited favorable responses from some users, negative reactions were more prevalent, particularly toward celebrity endorsements, excessive advertising exposure, and users’ relationships with sportsbook brands. Consistent with previous research (Deans et al., 2017; Lamont et al., 2016; Nyemcsok et al., 2021; Seal et al., 2022), music and humor seem to provide entertainment values to the audiences and make sports betting attractive, interesting, harmless and successful. However, the current findings also suggest that these strategies may backfire when perceived as intrusive or irresponsible, especially in the context of widespread ad saturation and sports betting promotion. Betting-related comments further underscored frustrations with sportsbook operators. Users described their unpleasant experiences with delayed withdrawals, frozen accounts, and misleading promotions, particularly around “free” or bonus bets. These findings indicate a discrepancy between the promotional messaging of sports betting advertising and users’ actual experiences with sportsbook platforms. When marketing messages emphasize excitement and accessibility but are not followed by relevant and timely services, they risk undermining credibility and trust. In this sense, the tactics designed to attract users may ultimately prompt public skepticism toward the industry and highlight the need for more responsible and transparent marketing practices.

As noted in sports marketing literature, this study also found the role of celebrity endorsements important. YouTube commenters expressed their mixed feelings about their endorsement. Well-known athletes, actors, comedians were used as brand ambassadors to enhance brand image and appeal. Among the most frequently mentioned celebrities, Charles Barkley and Paige Spiranac were evaluated relatively positively, with users praising Barkley’s humor and Spiranac’s physical attractiveness. In contrast, Wayne Gretzky, Jamie Foxx, and the Manning family received harsh criticisms with commenters describing them as “greedy” or “shameful” for endorsing gambling.

These findings align with prior research indicating that celebrity endorsements in gambling and other high-risk products are popular but often double-edged. While celebrities can capture attention and strengthen brand associations, they also risk a loss of credibility (Hing et al., 2014; Pitt et al., 2024). Authenticity and congruence between the celebrity’s persona and the product category are crucial determinants of persuasive effectiveness (Erdogan, 1999; McCracken, 1989). However, this connection seemed invalid for sports betting promotions, as endorsers were often criticized for legitimizing harmful behaviors and for appearing overly commercialized. Our findings extend this literature by showing how these dynamics manifest in online discourse. YouTube commenters explicitly questioned the sincerity and ethics of high-profile endorsers, suggesting that celebrity appeal in gambling advertising may invite heightened scrutiny rather than universal admiration. This tendency highlights the reputational risks that endorsers might face when lending their image to gambling promotions and underscores the importance of authenticity and responsibility in celebrity-driven marketing strategies.

Sportsbook brands themselves were another important topic of discussion. BetMGM, DraftKings, and FanDuel received the most mentions but were evaluated less positively than smaller brands such as PointsBet, Barstool, and Caesars, which attracted proportionally more favorable feedback. As noted, the visibility of these top three brands might be also due to their higher advertising exposures. Users often criticized the leading brands for poor customer service, delayed withdrawals, excessive advertisements, and misleading promotions, emphasizing dissatisfaction with brand–consumer relationships rather than with technical efficiency or usability of the apps. These findings suggest that trust, transparency, and responsiveness are central to bettors’ perceptions of sportsbook operators.

These findings suggest implications for advertisers, sportsbook operators, and regulators. For advertisers, the prevalence of negative and skeptical sentiment toward sports betting promotions highlights the importance of socially responsible messaging and audience trust. Prior research has shown that advertising fatigue and perceived moral irresponsibility can heighten resistance to gambling and alcohol marketing (Binde, 2014; Hing et al., 2023). Therefore, advertisers should prioritize authenticity, moderation, and responsible gambling cues rather than relying on celebrity appeal or humor alone (Thomas et al., 2023). Also, even if using celebrities is effective in getting people’s attention, having them in anti-sports betting campaigns or risk-messaging about gambling might be a good effort toward responsible sports betting promotion (Pitt et al., 2024). Identifying an optimal frequency for sports betting advertisements and incorporating clear responsible-gambling messages may help balance promotional engagement with ethical accountability.

For sportsbook operators, transparency, fairness, and service reliability appear central to shaping public attitudes. Promotions such as bonus bets or free bets have widely used as marketing strategies (Hing et al., 2024), yet YouTube commenters often perceived them as deceptive or overly complex, which echoes previous findings on consumer distrust in gambling promotions (Lopez-Gonzalez & Griffiths, 2018). Clearer communication of promotional conditions, timely withdrawal processing, and consistent customer service may strengthen trust and retention.

Regulatory bodies also have an important role in fostering responsible industry standards. Consistent with prior scholarship (Pitt et al., 2024), public sentiment in this study supports stricter oversight of advertising volume and celebrity endorsement. Lessons may be drawn from other jurisdictions, such as Italy’s 2018 ban on gambling advertisements, the United Kingdom’s restrictions on celebrity endorsers (A Closer Look, 2024; Hernandez, 2022), and Australia’s call for tighter regulations to protect children and minors (Pitt et al., 2024). U.S. regulators and self-regulatory agencies could adopt clearer guidelines on promotional transparency and withdrawal policies to mitigate public distrust of sports betting. We acknowledge that implementation will be challenging given fragmented legal authority, jurisdictional loopholes, and the rapid evolution of AI-mediated and social media advertising ecosystems (Parker et al., 2024). Accordingly, interagency coordination, tighter guidance on digital advertising and AI use, and periodic review to close legal loopholes may be necessary as platforms and marketing technologies continue to evolve.

As the legalization of sports betting continues to expand across the U.S., public perceptions and attitudes are likely to evolve with increased promotional activities. The convenience of online betting through smartphones and the visibility of real-time advertising may normalize betting behaviors, but this normalization also risks fostering negative attitudes if consumer frustrations and gambling-related harms are not properly addressed. Balancing entertainment with responsible gambling will be essential for the industry’s long-term sustainability. The history of restrictions on cigarette advertising, including the ban on television and radio commercials, offers a cautionary parallel for what can occur when industries fail to respond to public and regulatory concern. Future research could examine how regulatory changes, advertising frequency, and responsible gambling messaging influence public sentiment over time. Cross-platform analyses, experimental studies, and focus group interviews with diverse consumer segments could also clarify how message tone, celebrity involvement, and brand transparency influence perceptions of legitimacy or harm of sports betting promotions.

This study has several limitations. First, the findings are based solely on YouTube comments, reflecting the perspectives of a vocal and self-selected subset of viewers rather than the broader population. Therefore, the results should be interpreted as reflecting expressed audience sentiment in online discussions rather than fully representative public attitudes. In addition, as with most research based on social media data, the authenticity of user-generated comments could not be fully verified. Although the dataset was collected directly from YouTube and filtered for duplicates and non-English entries, it remains possible that some comments were produced by automated or bot accounts rather than human users. Second, prior literature on consumer reviews indicates that extreme online reviews are disproportionately prevalent, as individuals are more likely to post when they hold strongly positive or negative views (Brandes et al., 2022; Schoenmueller et al., 2020). These biases may have influenced the sentiment distribution, amplifying dissatisfaction relative to more moderate perspectives on sports betting promotions or operators. Third, the keywords used to identify sports betting videos on YouTube were selective. Even though we carefully examined various lists of major sports betting operators ranked in the U.S. to guide keyword selection, potential biases remain because comments on videos not retrieved with these keywords were excluded from the analysis. Finally, demographic analyses were not possible due to YouTube’s restrictions on user data disclosure, which limit access to information such as age, gender, and socioeconomic status.

Future research could address these limitations by employing longitudinal or cross-platform analysis to examine how sentiment patterns evolve over time and across different media environments. Expanding analysis to other social platforms such as X (Twitter), TikTok, or Reddit may reveal how diverse audiences and platform features influence the tone and dynamics of sports betting discourse.

## Data Availability

The datasets used and analyzed during the current study are available from the corresponding author on reasonable request.
